# Using Human Biomonitoring Data to Support Risk Assessment of Cosmetic Ingredients—A Case Study of Benzophenone-3

**DOI:** 10.3390/toxics10020096

**Published:** 2022-02-19

**Authors:** Christophe Rousselle, Matthieu Meslin, Tamar Berman, Marjolijn Woutersen, Wieneke Bil, Jenna Wildeman, Qasim Chaudhry

**Affiliations:** 1 European and International Affairs Department, Anses, 94701 Maisons-Alfort, France; christophe.rousselle@anses.fr; 2 Risk Assessment Department, Anses, 14 rue Pierre et Marie Curie, 94701 Maisons-Alfort, France; 3 Ministry of Health, Jerusalem 9101002, Israel; tamar.berman@moh.gov.il; 4 RIVM National Institute for Public Health and the Environment, 3721 MA Bilthoven, The Netherlands; marjolijn.woutersen@rivm.nl (M.W.); wieneke.bil@rivm.nl (W.B.); jennawildeman@outlook.com (J.W.); 5 Department of Clinical Sciences and Nutrition, University of Chester, Parkgate Road, Chester CH1 4BJ, UK; q.chaudhry@chester.ac.uk

**Keywords:** human biomonitoring, UV filters, benzophenone-3, HBM4EU, risk assessment, RCR, margin of safety

## Abstract

Safety assessment of UV filters for human health by the Scientific Committee on Consumer Safety (SCCS) is based on the estimation of internal dose following external (skin) application of cosmetic products, and comparison with a toxicological reference value after conversion to internal dose. Data from human biomonitoring (HBM) could be very useful in this regard, because it is based on the measurement of real-life internal exposure of the human population to a chemical. UV filters were included in the priority list of compounds to be addressed under the European Human Biomonitoring Initiative (HBM4EU), and risk assessment of benzophenone-3 (BP-3) was carried out based on HBM data. Using BP-3 as an example, this study investigated the benefits and limitations of the use of external versus internal exposure data to explore the usefulness of HBM to support the risk assessment of cosmetic ingredients. The results show that both approaches did indicate a risk to human health under certain levels of exposure. They also highlight the need for more robust exposure data on BP-3 and other cosmetic ingredients, and a standardized framework for incorporating HBM data in the risk assessment of cosmetic products.

## 1. Introduction

UV filters are used in sunscreen and other cosmetic products to protect the skin by absorbing or reflecting potentially harmful UVA and UVB rays. 

UV filters in sunscreens are regulated in Europe in the Cosmetic Product Regulation (CPR), which provides a broad regulatory framework for restricting chemicals with potential risks to human health. The CPR does not include specific restrictions on endocrine disrupting (ED) chemicals, however, and as there was concern for these substances the European Commission started a review of cosmetic ingredients with ED properties in 2018 [1]. As a result of the review, the Commission established a “short” list of potential EDs in cosmetics, which included benzophenone-3 (BP-3). Subsequently, the Scientific Committee on Consumer Safety (SCCS), which provides opinions on health and safety risks (chemical, biological, mechanical and other physical risks) of non-food consumer products (cosmetics, personal-care products, toys, textiles, etc.), was mandated by the Commission to carry out a safety assessment of BP-3 in view of concerns related to potential ED properties [2].

The SCCS approach to assessing the safety of cosmetic ingredients to the consumers is based on first estimating the external dose that is in contact with the skin, followed by estimation of the internal dose that will be systemically available after crossing the skin. The internal exposure dose is then compared to a reference value that is either derived from human or experimental data. This toxicological reference value (TRV) is used by the SCCS to calculate a margin of safety (MoS) [3]. In this context, human biomonitoring (HBM) can provide a very useful tool because it provides real-life internal exposure of the human population to a given chemical in terms of measured concentrations in human samples, such as blood and urine. There has been an increasing use of HBM for exposure assessment in the European risk assessment schemes in recent years, although the level of use is still limited. Based on a survey of different risk assessment frameworks in the EU, less than 30% of respondents replied that HBM is regularly applied in the regulatory domain in their country [4]. The use of HBM in risk assessments has also been uneven across different frameworks; for example, compared to pesticides, biocides, and heavy metals, the use of HBM data has been scarce in cosmetics safety assessments.

The European Human Biomonitoring Initiative (HBM4EU), running from 2017 to 2022, has established a European-Union-wide human biomonitoring program to generate knowledge on human internal exposure to chemical pollutants and their potential health impacts in Europe, to support policy makers’ efforts to regulate chemical safety and improve public health in Europe [5]. One of the goals of HBM4EU is to support policy making by providing evidence of the actual exposure of EU citizens to chemical substances and mixtures to inform risk assessment. The current study was aimed at exploring the potential usefulness of HBM data for supporting the risk assessment of cosmetic ingredients, using BP-3 as an example.

## 2. Methodological Approach

### 2.1. Risk Assessment Based on External Approach

The approach used by the SCCS to assess the safety of cosmetic ingredients, for which an opinion is required, is based on the calculation of a margin of safety. The method followed by the committee is described in detail in the Note of Guidance [3].

#### 2.1.1. Margin of Safety Calculation

In the risk characterization of a cosmetic ingredient, the main focus is on systemic effects, with separate consideration of any local effects. The MoS is generally calculated based on oral toxicity studies unless robust dermal toxicity data are available. For this, a toxicological point of departure (PoD), derived from oral studies in test animals, has historically been used in the following Equation (1):

MoS = PoDsys/SED(1)

where PoDsys (systemic PoD in mg/kg bw/day) is the potency descriptor, preferably a benchmark dose (BMD) or alternatively a NOAEL (no observed adverse effect level) or LOAEL (lowest observed adverse effect level) from a repeated-dose toxicity study. As these values are often based on oral studies, they are corrected for the oral bioavailability of the compounds to derive the PoDsys. It is considered that not more than 50% of an orally administered dose is systemically available. Thus, in the absence of data, 50% of the administered dose is used as the default oral absorption value for a cosmetic ingredient and the PoDsys is derived from the PoD by dividing by 2. SED (systemic exposure dose in mg/kg bw/day) is a dose descriptor for the systemic exposure to the substance calculated as the amount of the substance systemically absorbed from cosmetic products. 

Taking into account the extrapolation uncertainties corresponding to differences in toxicokinetics and toxicodynamics between experimental animals and humans (a factor of 10), and toxicological sensitivity differences within the human population due to differences in biology, age, sex, health status, etc. (a factor of 10), an MoS equal to or higher than 100 (10 × 10) is considered an adequate indicator of safety of the substance to human health. Other default assessment factors (AFs) may also be applied to take into account for example lack of data, but this was not applicable in this case [6].

#### 2.1.2. Hazard Assessment: Selection of an External Critical Dose

In this study, the selection of the critical effect, the key study, and the external critical dose of BP-3, was carried out following the methodology described in the SCCS Notes of Guidance [3]. Cosmetic safety assessments have historically used NOAEL as the toxicological PoD. The NOAEL is the highest dose or exposure level where no (adverse) treatment-related effects were observed. The external critical dose of the assessed ingredient (e.g., BP-3) is usually derived from a repeated-dose toxicity study that indicates an adverse effect, such as a chronic or a reproductive toxicity study [3].

The study selection process considered the assessment of all toxicological effect(s), their dose-response relationships, and possible threshold(s). Moreover, all periods of exposure including the most sensitive ones were considered, and in particular, exposure during pregnancy. The studies in which pregnant animals were exposed were therefore more thoroughly assessed.

#### 2.1.3. Exposure Dose (SED) Calculation Based on Skin Application

The human exposure to BP-3 can arise from cosmetic products as well as from other sources. BP-3 is currently regulated in sunscreens as a UV-filter with a maximum allowed concentration of 6% and in other cosmetic products with a concentration limit of 0.5%. BP-3 is also used in other product groups, such as coating products, plasters, modeling clay and finger paints, machine washing liquids/detergents, automotive care products, paints and coating or adhesives, and fragrances and air fresheners [7]. Exposure to BP-3 used as a UV-filter in cosmetic products was calculated based on the exposure scenarios by multiplying the concentration of BP-3 at the maximum approved concentrations (6% in sunscreen products and 0.5% in other cosmetic products). The external dermal exposure (E_dermal_) per day of BP-3 was then calculated taking into account the retention factor, concentration of BP-3 in the product, and amount of product applied daily, according to Equation (2):

E_dermal BP-3_ = C_BP-3_ × q_x_ × f_ret_ (2)

where E_dermal BP-3_ (mg/day): external exposure to BP-3 available for dermal uptake from a product category. 

C_BP-3_ (mg/g): concentration of BP-3 in the product category.

q_x_ (g/day): amount of product category that is applied per day.

f_ret_: retention factor specific to the product category; for leave-on cosmetics, including UV-cream, a fraction of 1 (100%) is used, while for rinse-off cosmetics (e.g., shower gel, shampoo, etc.) a smaller fraction is used that depends on the type and use of the respective product.

Next, external dermal exposure was multiplied by skin penetration value to calculate the systemic exposure dose (SED). Skin penetration can be determined from in vivo or in vitro studies. The approach preferred by the SCCS is based on selecting in vitro studies using published criteria [8].

### 2.2. Risk Assessment Based on Internal Approach

In HBM the internal human exposure is determined by measuring a substance itself or its metabolites in biological samples, usually blood or urine, in a human study population. The risk is calculated by comparing this internal exposure to a human biomonitoring guidance value (HBM-GV). As the HBM-GV used for BP-3 has no legal status, and has not been derived as an official HBM4EU guidance value, it should be considered a provisional HBM-GV in the context of this study. 

#### 2.2.1. Risk Characterization Based on HBM Data

Following the internal approach, the risk characterization ratio (RCR) for BP-3 is calculated by comparing the typical case (TC) exposure estimate and the reasonable worst-case (RWC) exposure estimate in each study to the provisional HBM-GV, as described in Equations (3) and (4):

(3) $$\mathrm{RCR}\;\left(\mathrm{TC}\right)=\frac{\mathrm{Range}\;\mathrm{of}\;\mathrm{median}\;\mathrm{values}}{\mathrm{provisional}\;\mathrm{HBM}-\mathrm{GV}}$$

(4) $$\mathrm{RCR}\;\left(\mathrm{RWC}\right)=\frac{\mathrm{Range}\;\mathrm{of}\;P95\;\mathrm{values}}{\mathrm{provisional}\;\mathrm{HBM}-\mathrm{GV}}$$

RCRs greater than 1 imply that the population assessed is (partly) exposed above the derived guidance value, and is thus potentially at risk of adverse effects from the chemical (e.g., BP-3).

#### 2.2.2. Provisional HBM-GV Derivation Using the Urinary Mass Balance Approach

A provisional HBM-GV is based on a PoD, reflecting the critical endpoint identified in hazard characterization and used as a reference point for the urinary concentration levels measured. For the derivation of the provisional HBM-GV, the urinary mass balance approach is applied [9]. This method can be applied to substances that are primarily eliminated through urinary excretion, and for which regular repeated exposure is likely. For such substances, the approach assumes a balance between the substance intake and the substance excretion, reaching a steady-state in the urine matrix. Under this assumption, the urinary excretion rate is a constant fraction of the intake rate [10].

Using this method, the critical dose from an experimental animal study (animal PoD) was transformed into a human biomarker concentration consistent with that dose (provisional HBM-GV) based on the urinary mass balance assumption. The animal PoD was first extrapolated to a human equivalent (external) TRV with application of assessment factors to account for extrapolation uncertainties (see Equation (5)). As for the external approach described above, the factors for allometric scaling and remaining toxicokinetic differences were applied to account for the differences in toxicokinetics and toxicodynamics between experimental animals and humans. An intraspecies factor was also applied [6].

(5) $$\mathrm{TRV}=\frac{\mathrm{Animal}\;\mathrm{PoD}}{\mathrm{Overall}\;\mathrm{AF}}$$

Next, a provisional HBM-GV was calculated from the TRV, using Equation (6), with parameters defined as follows:

- provisional HBM-GV: the human biomonitoring guidance value below which no adverse health effect should be expected, expressed as the substance concentration per gram creatinine (µg/g creatinine).
- TRV: toxicological reference value, the external human value corresponding to the animal PoD (mg/kg bw/day).
- Fue: substance-specific steady-state fraction of urinary excretion, the daily proportion of the intake dose excreted in urine.
- Creatinine excretion rate adjusted to BW: typical 24 h creatinine excreted, adjusted to default human bodyweight (g/kg bw/day), to compensate for differences in the volume of urine excreted.

(6) $$\mathrm{provisional}\;\mathrm{HBM}-\mathrm{GV}\;=\frac{\;\mathrm{TRV}\;*\;\mathrm{Fue}\;\left(\mathrm{substance}\right)}{\mathrm{Creatinine}\;\mathrm{excretion}\;\mathrm{rate}\;\mathrm{adjusted}\;\mathrm{to}\;\mathrm{BW}}$$

#### 2.2.3. Systemic Exposure Dose Calculation Based on an Internal Approach 

A systematic review was performed to assess the levels of BP-3 exposure in the European general population using the search engine Embase.com (Amsterdam: Elsevier) to gather all available human biomonitoring studies. Articles were qualitatively considered for compiling the BP-UV filter exposure database with European exposure data. Selected articles were then quantitatively screened for meta-analysis of BP-3 exposure in the European general population. As older studies are considered less relevant for the current situation, only data after 2006 were included. The analysis included only European studies that applied enzymatic deconjugation of the samples and storage at <4 °C, as these are generally considered critical aspects of sample treatment to obtain reliable and comparable results [11]. 

After study selection based on the criteria, the overlapping cohorts amongst the multiple included articles were removed. In filtering out the overlapping cohorts, studies with larger sample sizes, more detailed data reporting, and analysis of multiple benzophenones, were prioritized. The risk of bias of the individual studies was evaluated and studies were excluded from the meta-analysis in case of a considerable risk of bias. 

The summary statistics were calculated for a typical case exposure and a reasonable worst case exposure. The typical case exposure is described by the median and range (min-max) of median values from the included studies and the reasonable worst-case by the median and range (min-max) of P95 values. Descriptive statistics that were below LOD or LOQ were replaced with the corresponding LOD or LOQ value, conforming to the statistical analysis of the CONTAM panel [12]. All statistical analyses were performed in Excel.

## 3. Results

### 3.1. Risk Assessment Based on External Approach

#### 3.1.1. Selection of External Critical Dose

Previous safety assessments of BP-3 by the European Scientific Committees [13,14,15] concluded that BP-3 can be safely used as a UV-filter up to 6% in cosmetic sunscreen products and up to 0.5% in all types of cosmetic products although it has the potential for contact allergy and photoallergy. The recent assessment by the SCCS [2] considered previous evaluations, as well as new information, with a particular focus on the potential ED properties of BP-3.

The information available under REACH has categorized BP-3 as non-sensitizing, non-irritant to eyes and skin, and suggests no acute adverse effects [7]. Both human epidemiological and animal experimental studies have associated BP-3 exposure to reproductive and developmental toxicity [2]. Within the CLP regulation (EC No. 1272/2008) (classification, labeling, and packaging) [16], BP-3 is not considered to meet the criteria for reproductive toxicity classification [7]. Systematic reviews have highlighted a varying relationship between BP-3 exposure and estrogenic, androgenic, and antiandrogenic activities frequently reported in vivo and in vitro [17,18]. However, the clinical relevance of the endocrine effects in these studies is not always clear, and uncertainty remains over when the observed endocrine effects can be interpreted as adverse. This led the SCCS to conclude that the currently available evidence on ED properties of BP-3—including in silico modeling and in vitro and in vivo studies—was inconclusive and at best equivocal when considered individually or taken together. Therefore, the SCCS did not derive a new PoD on ED properties to use in the risk assessment. 

To calculate the MoS, the SCCS used the lowest NOAEL values (67.9 mg/kg bw/d) derived from a study by Nakamura et al. [19]. The Nakamura et al. (2015) study [19] determined the effects of maternal and lactational exposure to BP-3 (between 0–3448 mg/kg/day) on the development and reproductive organs of the offspring of time-mated female rats. There were no significant differences on the reproductive/ pregnancy parameters or sex ratio of the offspring between the control and BP-3-dosed groups. In the highest dose groups, there was a significant reduction in the normalized anogenital distance on post-natal day (PND) 23 in male pups, a decrease in serum ALT (albumin transferase) and a significant increase in cholesterol levels in both female and male offspring at PND 23. Body, ovarian and uterine weights were significantly reduced. Relative liver-to-body-weight ratios were also significantly increased and paired kidney weights were lower. 

In addition, in the highest dose group, seminiferous tubules contained few or no spermatocytes compared to the control group. The SCCS considered 3000 ppm as the LOAEL and 1000 ppm (67.9 mg/kg bw/day) as the NOAEL based on the decrease in spermatocytes. This value was used as a PoD for risk characterization.

#### 3.1.2. Systemic Exposure Dose (SED) Calculation Based on an External Approach

- Skin penetration

Dermal absorption was determined using an in vitro study with 6% BP-3 that had been considered in the previous SCCP Opinion [15]. Due to some shortcomings in this study, the SCCS applied an additional 2 × SD (standard deviation) to the reported mean dermal absorption value. A dermal absorption of 9.9% was used for the calculation of SED. For other cosmetic products, a dermal absorption of 8% based on the results of the study performed with 2% BP-3 was used [2].

- Calculation of the SED

The calculation of the SED following application of a whole-body UV-cream containing up to 6% BP-3, the intended level of use requested by the cosmetic industry, was estimated at 1.78 mg/kg bw/day, as detailed in Table 1. 

Exposure via other types of cosmetic products (UV or non-UV) by dermal, inhalation or oral routes was also considered by SCCS (see Table 2 below).

#### 3.1.3. MoS Calculation

Because of the evidence for rapid and almost complete absorption of BP-3 from the oral route, the SCCS did not apply any adjustment for bioavailability to this NOAEL value. Details of the calculation of systemic exposure dose (SED) are presented in the Tables in Annex 2 of the SCCS opinion. The calculation of MoS for different product types ranged between 1257 for lipstick and 36–38 for UV aerosolized spray and cream. MOS for hand cream and face cream were 317 and 447, respectively (see Table 2). 

A margin of safety (MoS) of 38 was estimated for the use of BP-3 at a concentration of 6% as a UV-filter in sunscreens for the whole-body application (in the form of cream/lotion or pump spray), 36 for sunscreen propellant spray, and 447 for the face, 317 for the hand and 1257 for lipstick application. The MoS was estimated at 585 for the use of BP-3 at 0.5% to protect cosmetic formulations against sunlight. As mentioned before, a default value of 100 (10 × 10) accounting for inter- and intraspecies differences is generally acceptable, and as an MoS of at least 100 therefore indicates that a cosmetic ingredient is safe for use [3], the SCCS assessment of BP-3 concluded that:

- The use of BP-3 as a UV-filter up to a maximum concentration of 6% in sunscreen products is not safe for the consumer with the exception of face cream, hand cream, and lipsticks
- The use of BP-3 up to 0.5% in cosmetic products to protect the cosmetic formulation is safe for the consumer.

### 3.2. Risk Assessment Based on Internal Approach

#### 3.2.1. Provisional HBM-GV Derivation Using the Urinary Mass Balance Approach

In this study, the same POD of 67.9 mg/kg bw/day as selected by the SCCS [2] was used as a starting point in the derivation of a provisional HBM-GV that was used to calculate the risk based on the biomonitoring data. In accordance with the ECHA guidance R.8, assessment factors (AFs) for allometric scaling, remaining interspecies differences, and intraspecies differences were applied [6]. The guidance additionally prescribes an AF for exposure duration extrapolation, where appropriate. This AF is generally applied to account for differences in exposure between the experimental toxicity study and real-life situation. However, as in the study from Nakamura et al. (2015) [19], animals were exposed during gestation, which corresponds to a sensitive window of exposure, and no other AF were considered necessary to extrapolate from sub-acute to chronic exposure duration. In view of this, an overall AF of 100 was constituted from the following default AFs:

- Allometric scaling (rat to human):  4
- Remaining interspecies differences:  2.5
- Intraspecies differences:       10
- Duration extrapolation:        1
- Overall AF:     4 * 2.5 * 10 * 1 = 100

The Fue was obtained from two human experimental exposure studies [20,21]. Hayden et al. (1997) reported BP-3 levels in urine to be 1–2% of the initial dose 10 h after dermal application to nine healthy adult volunteers [20]. Sarveiya et al. (2004) similarly reported up to 1% BP-3 of the initial dose dermally applied to three female volunteers and measured in urine after 48 h [21]. It was therefore considered appropriate to incorporate an Fue of 0.01 (1%) as a conservative value. 

The HBM4EU default average daily creatinine excretion was incorporated in the derivation of the provisional HBM-GV. The HBM4EU default creatinine value is based on the calculation according to Aylward et al. (2009) [22], and amounts to an average total amount of creatinine excreted of 1.4 g/L per day for both sexes combined. Adjusted to the default body weight of 70 kg, as defined by ECHA R.8 [6] and followed by HBM4EU, this amounted to an average creatinine excretion rate of 0.02 g/kg bw/day. Table 3 presents an overview of the information and parameters incorporated in the provisional HBM-GV calculation.

Application of the overall AF to the animal PoD in Equation (7) provided the TRV:

(7) $$\mathrm{TRV}\;=\frac{67.9}{100}=0.68\;\mathrm{mg}/\mathrm{kg}\;\mathrm{bw}/\mathrm{day}$$

Incorporating the TRV and remaining parameters (Table 3) in Equation (8) provided the provisional HBM-GV:

(8) $$\mathrm{provisional}\;\mathrm{HBM}-\mathrm{GV}\;\left(\mathrm{GenPop}\right)=\frac{0.68\;*\;0.01}{0.02}=0.34\;\mathrm{mg}/g\;\mathrm{creatine}\;=340\;µg/g\;\mathrm{creatinine}$$

#### 3.2.2. Systemic Exposure Dose Calculation Based on an Internal Approach 

The internal exposure was assessed with a meta-analysis of the biomonitoring data. Screening and selection resulted in 17 articles with biomonitoring data on BP-3 in the European cohorts. After application of the exclusion criteria and selection of the studies that used sufficient numbers of samples (>120), 8 studies were selected. Out of these, only 3 studies that had used creatinine correction were considered in the meta-analysis [23,24,25]. Table 4 presents the cohort characteristics and study-specific descriptive statistics of creatinine-corrected concentrations of BP-3 and BP-1. Frederiksen et al. (2013) [25] reported BP-3 measurements in first morning urine samples of mother–child pairs in Denmark. Dewalque et al. (2014) [24] reported BP-3 measurements in spot urine samples from the general population in Belgium. Adoamnei et al. (2018) [23] reported BP-3 and BP-1 measurements in first morning urine samples of male students in Spain. However, this study did not report maximum measured concentrations.

The TC of BP-3 exposure (based on median values across the studies) ranged from 0.60 to 4.40 µg/g creatinine, with a median of 1.30 µg/g creatinine. The RWC BP-3 exposure (based on P95 values across the studies) varied between 16.30 and 392.00 µg/g creatinine, with a median of 33.00 µg/g creatinine. 

#### 3.2.3. Risk Characterization Ratio (RCR) Calculation

Comparing the TC exposure range (0.60 to 4.40 µg/g creatinine) and the RWC exposure range (16.30 to 392.00 µg/g creatinine) with the provisional HBM-GV (340 µg/g creatinine) in Equations (3) and (4) provided the following RCRs:

$$\mathrm{RCR}\;\left(\mathrm{TC}\right)=\frac{0.60\;\mathrm{to}\;4.40}{340}=0.002\;\mathrm{to}\;0.01=\mathrm{RCR}<1\;$$

$$\mathrm{RCR}\;\left(\mathrm{RWC}\right)=\frac{16.30\;\mathrm{to}\;392.00}{340}=0.05\;\mathrm{to}\;1.15=\mathrm{RCR}>1$$

For typical case exposure, the RCR was below 1, so median HBM results did not exceed the orientation value. However, for a reasonable worst-case exposure, the RCR slightly exceeded 1. Table 4 shows the HBM values exceeding the provisional HBM-GV in bold, as well as the RCR values at the RWC/P95 value. The P95 value reported by Frederiksen et al. (2013) [25] for Danish females exceeded the provisional HBM-GV. The maximum values for female participants and children in Frederiksen et al. (2013) [25] and for male participants in Dewalque et al. (2014) [24] exceeded the provisional HBM-GV.

In summary, HBM values did not exceed the provisional HBM-GV for a typical exposure scenario and were therefore within safe limits. They did, however, exceed the provisional HBM-GV slightly in the reasonable worst-case exposure scenario, indicating potential risk to the highly exposed part of the population. 

## 4. Discussion

In most chemical risk assessment frameworks, the default approach is to assess external intake from different sources of exposure, as well as via different routes of exposure, which are often assessed separately. In the context of cosmetics risk assessment, dermal exposure is calculated using the estimated amount of a product applied, retention factor of the product, and skin penetration value of the chemical. This approach includes various uncertainties and often overestimates the real uptake because default, conservative estimates are used, e.g., for the amount of product used. At the same time, actual (real-life) exposure may be underestimated by not taking into account that exposure to a chemical substance may occur from different sources that may fall under different legislative frameworks [4]. The use of HBM studies in chemical risk assessment has not yet become common practice, and its value in risk assessment remains largely unexplored. Within the HBM4EU project, it is the aim to increase the quality of HBM data and provide policymakers with tools to use these data in risk assessment. 

BP-3 is mainly used in cosmetics, and a recent safety evaluation has been performed by the SCCS, instigated by concerns on the ED properties of BP-3. As is usual practice, the exposure estimate in the opinion was based on the use patterns of sunscreen and other cosmetic products (SCCS/1625/20) and dermal absorption of BP-3. No risk assessment for exposure of the European population to BP-3 using HBM data has been performed to date. This study used BP-3 as an example to investigate the usefulness of HBM in risk assessment, and for informing decision-making throughout the risk assessment process—in particular to address the additional research questions. 

This study has compared two parallel risk assessment approaches—one based on the external exposure (the SCCS approach), and the other on internal exposure data from three different countries in Europe (the HBM approach). The results showed a remarkably similar overall conclusion in that the highly exposed individuals would be at a (slightly) increased risk from the intended levels of BP-3 use in certain product categories. 

It needs to be noted that the internal dose approach used in HBM includes all sources of exposure, including from cosmetics. Therefore, although the estimated RWC is only slightly above 1, it does not truly reflect the risk to consumers from cosmetics only. The RWC was based on the P95, which could reflect exposure to BP-3 via cosmetic products, whereas the RTC, which is based on the mean, could be more representative of the background exposure to BP-3 either via cosmetic products other than sun-care products or via other consumer products. BP-3 is indeed also used in consumer products to protect formulations from degradation by the sun. Unfortunately, in the biomonitoring studies that have measured BP-3 in Europe, there is no product use data to show where this exposure comes from. In the study from Zamoiski et al. (2016) [26], the authors observed based on urinary concentrations of BP-3 measured in NHANES 2003–2006 and 2009–2012 that BP-3 was positively associated with self-reported frequency of sunscreen use and that concentrations of BP-3 in the more frequent users were 116.8 µg/g creatinine, which is above the P95 reported in Belgium and Spain [23,24] but lower than the Danish values [25]. It was rather surprising to see the highest BP-3 exposure in a study with a sampling period only in the fall in a northern country [25]. A similarly unexpected outcome was seen in a recent study performed in Israel, where sunscreen products are widely used, that reported relatively low internal exposure [27]. Thus, more information on product use would be valuable to gain more insight on the products contributing to BP-3 exposure. Moreover, the study populations were relatively small, which adds uncertainty in the extrapolation to the entire European population. Vogel et al. (2019) [28], recommended samples of at least 72 to 120 individuals to be able to define reference values for the general population, which is in line with the 3 biomonitoring studies we have included in our assessment. 

In contrast, the external approach, as currently used by the SCCS, is conservative as it considers that the concentration is at the maximum requested level for all the cosmetic products formulated with BP-3. In addition, for the aggregated exposure scenario, the SCCS does not take into account market penetration and assumes that all the products in the product category concerned contain BP-3. Neither of these assumptions are realistic and they make the SCCS approach conservative. Where data are available, the SCCS methodology incorporates a refinement in terms of the use of a probabilistic approach to exposure assessment to take into account the distribution of values for exposure parameters, such as concentration in the products, amounts of the products used, etc. However, this approach is subject to the availability of data on these distributions and therefore may not be practicable in all cases.

There are certain differences between the external and internal dose approaches that also warrant further attention. 

Both external/internal exposure-based approaches have certain advantages and disadvantages that are illustrated in Table 5.

HBM data provide data on exposure from all routes and sources, which can be an advantage when there is a concern over aggregated exposure. In such situations, the availability of actual data is a distinct advantage because aggregate exposure measurements often suffer from high levels of uncertainty. However, to answer questions on specific product contributions, additional product-specific exposure estimations will remain a necessity. Based on the available information, it is difficult to have a clear understanding of the exposure profile to BP-3 and the contribution of different sources of exposure. BP-3 is used as a UV-filter not only in cosmetic sun-care products, but also in other consumer products such as washing and cleaning products, anti-freeze products, biocides (e.g., disinfectants, pest control products), and printing inks in food contact materials [7]. Intervention studies have shown that by avoiding the use of cosmetic products containing BP-3, internal exposure can be significantly reduced, which indicates that cosmetics are an important source of exposure to BP-3 [29,30]. 

Another consideration for biomonitoring studies is that they are by nature retrospective. They can only be used for retrospective risk management by showing the actual exposure under a certain regulatory regime, but not to predict exposure before placing a new product on the market, or authorizing a new ingredient. HBM data can, however, be used to assess the impact of new regulatory measures by measuring time trends. In fact, the HBM data used here were from before the previous decrease in maximum concentration of BP-3 in cosmetic products that went into effect in 2017. An update of this risk assessment will be performed within HBM4EU based on measurements from 2020, the outcome of which will give a better understanding of the current situation. 

Where there is a special interest in a specific, vulnerable groups within the population, such as children or pregnant woman, HBM data have the advantage in terms of providing an insight into the exposure of separate groups. This is equally possible through modeling of external exposure, but only if the required use data are available for these groups. 

The SCCS guidance for the testing of cosmetic ingredients and their safety evaluation [3] noted that HBM data should be used to support risk assessment and risk management. For example, HBM can serve to evaluate whether the dose estimation model over- or under-estimates the real exposure. Especially in light of the prohibition of in vivo animal studies on cosmetic substances, HBM offers a means to inform risk assessment by providing real-life in vivo information from human studies, without the need for interspecies extrapolation or the limitation of a small number of subjects often used in human volunteer studies. Indeed, HBM data were also noted in the SCCS opinion on BP-3 (SCCS/1625/20), but not used as such in modeling the exposure, or supporting the safety assessment.

## 5. Conclusions

HBM has the potential to be a useful tool for regulatory risk assessment as it provides actual exposure levels, but it is currently rarely applied in regulatory practice. Within the HBM4EU project, it was proposed to collect and create successful examples on the use of HBM data in chemical risk assessment. The experience from these cases could be used as a basis for developing harmonized guidance on the use of biomonitoring data in risk assessment, which was indicated as critical to the use of HBM. The aim of this work was to compare an HBM case study on BP-3 with a ‘traditional’ risk assessment. The results have shown that both the current SCCS approach and the HBM approach have their own advantages for risk assessment of cosmetic products. In the case of BP-3, both approaches indicated similar levels of risk. The comparison also indicated that HBM data can be useful in supporting risk assessment by providing real-life data on exposure, and may also play an important role in post-approval assessment studies on exposure trends. However, before being adopted for use on a regular basis in regulatory risk assessments, more efforts are needed to better harmonize HBM surveys, and to obtain robust data that are representative of the exposure of the European population. This study has also highlighted the need for the development of a standardized framework for incorporation of HBM data in the current risk assessment of cosmetic products. 

## Figures and Tables

**Table 1:** Systemic exposure by dermal route from whole-body UV-cream containing up to 6% BP-3 (adapted from SCCS opinion [2]).

Description	Parameter	Value	Unit
Amount of sunscreen product used	A	18	g/day
Concentration of BP-3	C	6	%
Dermal absorption	DA_p_	9.9	%
Human body weight	Bw	60	kg
Systemic exposure dose (SED)	A × 1000 mg/kg × C/100 × DA_p_/100/bw	1.78	mg/kg bw/day

**Table 2:** Calculation of margin of safety for different products containing BP-3 (adapted from SCCS opinion [2]).

Product Categories	Conc.	Surface	Total * Systemic Exposure Dose (SED) mg/kg bw/d	NOAEL **	MoS
**UV cream**	6%	whole body	1.78	67.9	38
**UV aerosolized spray**	6%	whole body	1.89	67.9	36
**UV pump spray**	6%	whole body	1.78	67.9	38
**UV face cream**	6%	face	0.15	67.9	447
**UV hand cream**	6%	hand	0.21	67.9	317
Non-UV ***	0.50%	whole body	0.12	67.9	585
**Lipstick**	6%	lips	0.05	67.9	1257

* Total = dermal + inhalation + oral, ** derived from prenatal and postnatal developmental study, oral, rat [19]. *** to protect cosmetic formulations.

**Table 3:** Overview of parameter values incorporated in the provisional HBM-GV calculation, with sources.

Parameter	Value	Source
Animal PoD	NOAEL = 67.9 mg/kg bw/day	SCCS opinion (2021) [2]
Overall AF	AF = 100	ECHA R.8 guidance, SCCS opinion [6]
Fue	1% or 0.01	Hayden et al. (1997); Sarveiya et al. (2004) [20,21]
24 h creatinine	0.02 g/kg bw/day	HBM4EU default from Aylward et al. (2009) [22]

**Table 4:** Cohort characteristics and descriptive statistics reported by the final three studies included in the meta-analysis of exposure. All concentrations (P50, P75, P95, max) are creatinine-corrected and thus expressed as µg/g creatinine. Concentrations > provisional HBM-GV in bold.

Study	Sampling Period	N/Sex	Age Range	Sample	Chemical	LOD (%>LOD)	P50	P75	P95 (RCR)	Max
Frederiksen et al. (2013) Denmark	September 2011–December 2011	143 M/F	6–11	Morning	BP-3	0.07 (97.0%)	2.00	6.30	33.00 (0.1)	**408.00**
		154 F	31–52	Morning	BP-3	0.07 (98.0%)	4.40	15.00	**392.00 (1.15)**	**2139.00**
Dewalque et al. (2014) Belgium	January 2013–April 2013	123 M	2–75	Spot	BP-3	0.20 (82.1%)	0.60	2.00	28.80 (0.08)	**414.20**
		138 F	1–85	Spot	BP-3	0.20 (83.3%)	1.30	4.40	33.30 (0.1)	141.30
Adoamnei et al. (2018) Spain	October 2010–November 2011	215 M	18–23	Morning	BP-3	0.20 (65.6%)	0.96	4.60	16.30 (0.05)	NA
					BP-1	0.10 (97.2%)	1.60	3.10	9.90 (0.03)	NA

N: sample size, M: male; F: female; age range: minimum-maximum age in years; LOD: limit of detection; NA: not available.

**Table 5:** Differences between current SCCS approach and HBM approach for risk assessment.

	Current SCCS Approach	HBM Approach
**Dose estimation**	Modeled/estimated	Measured, real-world conditions
**Exposure pathways**	Dermal exposure	Provides data on total exposure from all exposure pathways
**Temporality**	No time lag; can be used in a predictive approach	Time lag between exposure estimate and risk assessment; only retrospective; cannot be used in a prospective approach
**Product specificity**	Calculations per product type, combining several conservative parameters in a deterministic assessment may lead to overestimation	No product-specific data: aggregate exposure modeling needed to identify relative contribution of a product to the overall exposure
**Consideration for toxicokinetic aspects**	Generally uses in vitro studies for dermal absorption and historic animal studies for PoD and applies an AF to correct for animal–human differences	Considers biotransformation and elimination of the substance in humans, but requires appropriate timing of sampling
**Conclusion of risk assessment for BP-3**	Exposure at the intended use levels exceeds safe dose for whole-body cream and spray but not face or hand cream	Exposure exceeds safe dose in highly exposed individuals

## Data Availability

Not applicable.

## Abbreviations

AF: assessment factor, ALT: albumin transferase, BMD: benchmark dose, BP-3: benzophenone-3, BW: body weight, C: concentration, CPR: Cosmetic Product Regulation, CLP: classification, labeling, and packaging, ED: endocrine disrupting, E_dermal_: dermal exposure, EU: European Union, f_ret_: retention factor, F_ue_: steady-state fraction of urinary excretion, HBM: human biomonitoring, HBM4EU: European Human Biomonitoring Initiative, HBM-GV: human biomonitoring guidance value, LOAEL: lowest observed adverse effect level, LOD: limit of detection, LOQ: limit of quantification, MoS: margin of safety, NOAEL: no observed adverse effect level, PND: post-natal day, PoD: point of departure, PoDsys: systemic PoD, q_x_: quantity applied per day, RCR: risk characterization ratio, RWC: reasonable worst-case, SCCS: Scientific Committee on Consumer Safety, SD: standard deviation, SED: systemic exposure dose, TC: typical case, TRV: toxicological reference value.
